# Cat and/or Dog Ownership, Cardiovascular Disease, and Obesity: A Systematic Review

**DOI:** 10.3390/vetsci8120333

**Published:** 2021-12-17

**Authors:** Cristina S. Barroso, Kathleen C. Brown, David Laubach, Marcy Souza, Linda M. Daugherty, Melanie Dixson

**Affiliations:** 1 College of Nursing, University of Tennessee, Knoxville, TN 37996, USA; 2 Department of Public Health, University of Tennessee, Knoxville, TN 37996, USA; kcbrown@utk.edu (K.C.B.); dlaubach@utk.edu (D.L.); 3 College of Veterinary Medicine, University of Tennessee, Knoxville, TN 37996, USA; msouza@utk.edu; 4 Social Work Office of Research and Public Service, Knoxville, TN 37996, USA; lindad@utk.edu; 5 Libraries, University of Tennessee, Knoxville, TN 37996, USA; mallen31@utk.edu

**Keywords:** one health, cat ownership, dog ownership, cardiovascular disease, obesity

## Abstract

Pet ownership, the most common human–animal interaction, is believed to bestow positive health benefits onto pet owners. However, there is limited research on substantiating these assertions. The aim of this review was to systematically identify, evaluate, and summarize primary research on the relationship between cat and/or dog ownership and cardiovascular disease (CVD), type 2 diabetes (T2D), and obesity to inform future research on pet ownership and chronic disease. How pet ownership was defined/measured, and identification of the chronic disease variables and health behaviors most often measured were emphasized. Two researchers independently searched PubMed and Web of Science, where One Health literature are mostly likely to be indexed, for peer-reviewed literature on pet ownership and CVD, T2D, and obesity. A review of 4541 titles and abstracts for relevance resulted in 34 manuscripts eligible for full-text review. Two researchers assessed each eligible manuscript and extracted data only from those that met the inclusion criteria (n = 14). Ten studies on CVD, four studies on obesity, and zero studies on T2D met the study criteria. The CVD and obesity variables varied and were not well described. The relationship between pet ownership and CVD and obesity varied (positive, negative, mixed effects, and no effect). Generalizability lacked across all studies: most studies were with Non-Hispanic White populations. Other areas of weakness were quality of study outcomes and instrument validity. Operationalization of pet ownership varied (from no verification to confirmed pet registration). Integration of the evidence-based influence of the human–animal connection through pet ownership on CVD and obesity may make prevention, mitigation, and treatment strategies more robust.

## 1. Introduction

Chronic disease, a continuing or reoccurring health condition for a long period of time [1], is the leading cause of death and disability in the United States (USA) [2,3]. Cardiovascular disease (CVD), type 2 diabetes (T2D), and obesity are the most common chronic diseases in the USA [4]. Most of the nation’s annual health care expenditures, approximately $3.6 trillion, are for treatment of chronic diseases [4,5], which is about $11,172 per person [5]. Yet, most chronic diseases (i.e., CVD, T2D, and obesity) are preventable with changes in dietary habits, physical activity, and tobacco control. 

In the USA, 67% of households have at least one pet, including 63.4 and 42.6 million households with at least one dog and one cat, respectively [6]. It is believed that pet ownership, the most common form of human–animal interaction, bestows positive benefits related to health outcomes, wellbeing, and quality of life onto pet owners [7,8]. It has been anecdotally claimed that pets stimulate physical activity (i.e., dogs) and better psychological wellbeing, and influence dietary habits in their owners. However, explicit pathways for how this type of human–animal interaction (pet ownership) may influence chronic disease are still unclear.

Pet ownership literature is mostly on dog ownership studies, and the findings from these studies are mixed. Whereas some studies reported dog ownership to be associated with a reduced cardiovascular risk [9,10,11] and all-cause mortality [10,12], other studies reported no evidence of association [13,14]. Miyake et al. (2020) found no evidence of association between dog ownership and obesity [15], while Lentino et al. (2012) found that dog owners self-reported less diabetes than non-owners [16]. Variations in study findings may be attributed to differences in study populations (e.g., healthy adults versus older adults versus hospitalized adults), study design (e.g., observational versus intervention), and measures (health condition/disease outcomes: lipid levels, blood pressure, smoking status, etc.; pet ownership: current versus past owner) used. Even less is known about cat ownership and human health [17,18].

An exploration of the human–animal relationship (pet ownership) and chronic disease, more than just CVD, is warranted. Given that cats and dogs are the most common pets in the world, a focus on how these pets influence human health is of importance. This review systematically identified, evaluated, and summarized primary research on the relationship between cat and/or dog ownership and CVD, obesity, and T2D to inform future research on pet ownership and chronic disease. How pet ownership was defined/measured, and identification of the chronic disease variables and health behaviors most often measured were emphasized.

## 2. Materials and Methods

This study addressed two research questions. First, what are the effects of pet ownership (positive, negative, mixed, or no effect) on health outcomes for CVD, obesity, and T2D? Second, how were pet ownership and chronic disease measures/variables assessed? The authors used the PRISMA guidelines [19] to conduct this systematic review and registered the protocol with Prospero in September 2019 (approval received in April 2020; registration number: CRD42020149538). To their knowledge, no other systematic review on both cat and/or dog ownership and CVD, obesity, and/or T2D exists in English. The inclusion criteria were original research on cat and/or dog ownership that reported CVD, obesity, or T2D outcomes for humans (16 years or older) and was published in English from January 2000 to April 2021. Exclusion criteria were studies on exotic animals or animals besides cats and dogs, literature reviews, and grey literature. As this review used publicly available literature, this research was exempt from institutional review board evaluation.

**Figure 1:**
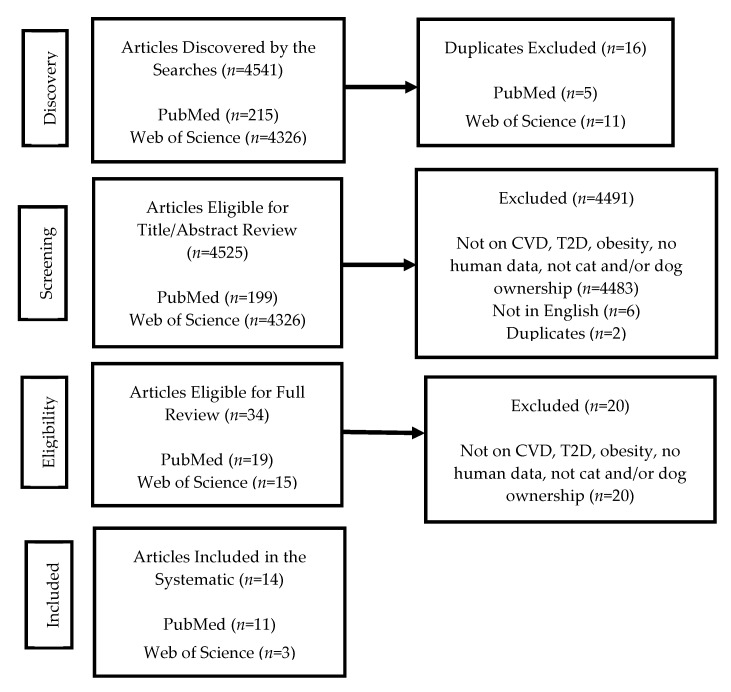
Flow diagram of studies identified and included in the systematic review.

To identify studies, a four-step strategy was used: discovery, screening, eligibility, and included (Figure 1). In the discovery step (June–December 2020), two researchers (CSB and KCB) independently used PubMed (including Medline; via National Library of Medicine) and Web of Science (Clarivate Analytics) to search for published literature, as One Health literature are most likely to be indexed in these search engines. A follow-up search was conducted in April 2021. Keywords and database-specific index terms used were (((chronic * OR heart OR cardiovas * OR “CVD” OR obes * OR overweight OR diabet *)) AND ((dog * OR cat * OR canine * OR feline *) AND ((pet OR pets)) AND (owner * OR companion * OR interact * OR bond * OR “human animal bond” OR “animal human bond” OR “animal assisted”))) AND (health * AND (impact * OR outcome * OR status OR effect * OR affect * OR consequen * OR result *)). “Chronic disease” was used as a search term to cast a wider net for potential manuscripts that focused on CVD, T2D, and obesity outcomes in humans. The searches resulted in 4541 journal articles: 215 from PubMed and 4326 from Web of Science. Sixteen duplicates were eliminated (PubMed: *n* = 5; Web of Science: *n* = 11), resulting in 4525 journal articles.

During the screening step, two reviewers (CSB and KCB), with a third reviewer (MS) to resolve discrepancies, independently performed a title/abstract review. Studies that were not original research, did not report on human CVD, T2D, or obesity outcomes, were not focused on cat and/or dog ownership (*n* = 4483), were not in English (*n* = 6), or were duplicates (*n* = 2) were eliminated. This resulted in 34 journal articles for full-text review.

In the eligibility step, all six authors conducted full-text reviews, two researchers per journal article. When there was disagreement, both researchers conducted a second assessment. If disagreement persisted, a third researcher reviewed the study, and a discussion was conducted to reach agreement. The full-text review resulted in 14 journal articles (*n* = 20 studies did not meet the inclusion criteria).

For each of the 14 studies (included step), two researchers independently extracted the study data: author, year, and title, purpose, study description, independent and dependent variables, CVD, obesity, or T2D measures, and operationalization of pet ownership), and main disease/condition results. A third reviewer (DL) consolidated the data and addressed discrepancies. Researchers also assessed each study for rigor using a quality/bias assessment tool developed by three team members (CSB, KCB, and LB) and CSB, KCB, or LB confirmed the assessment. Areas assessed were degree of pertinence to the research question, quality of outcomes, instrumentation validity, and generalizability (quality of comparison groups, sample size, and bias). The rating system (Supplementary Table S1) for each criterion ranged from 0 (unknown) to 3 (extremely well-explained and outlined). A summary score for each study was the sum of all quality/bias criteria (range: 0–12). The higher the summary score, the higher the study quality and the lower the study bias.

Data synthesis of the 14 articles consisted of a description of findings, trends, and gaps in the research. Articles were categorized according to the reported effect (positive, negative, mixed, or no effect) cat and/or dog ownership had on the main CVD, obesity, or T2D outcomes. Recommendations to further knowledge and practices are provided.

## 3. Results

Table 1 briefly summarizes the 14 studies in this review. For a full description of the 14 included studies, see Supplementary Table S2. Ten studies on CVD [20,21,22,23,24,25,26,27,28,29], four studies on obesity [30,31,32,33], and zero studies on T2D met the criteria. The 10 CVD studies were conducted in Australia (two studies) [20,26], China [29], Croatia [28], England [21], Sweden (two studies) [23,24], and the USA (three studies) [22,25,27]. Common CVD variables were body mass index (BMI) [20,25,27,29], systolic blood pressure [20,22,27], diastolic blood pressure [20,22], high-density lipoprotein (HDL) cholesterol [20,22], low-density lipoprotein (LDL) cholesterol [20,22], physical activity [20,25,29], smoking status [21,22,25,27,29], and alcohol consumption [20,21,25,29]. Three obesity studies were conducted in the USA [30,31,33] and one in the Netherlands [32]. The obesity variables were BMI [30,31,33], body weight [32], fat percentage [32], waist circumference [32], health status [30], dietary intake [30,31], physical activity [30,31,33], stress [33], and social support [31,33].

### 3.1. CVD Studies

Sample sizes for the CVD studies ranged from n = 59 (male participants only) [28] to *n* = 3,432,153 [23] and most were conducted with middle-aged and/or older adults; ages ranged from 40 to 84 years in four studies [20,23,24,25] and three studies did not mention a specific age but focused on diseases with onset later in life [22,26,28]. One study included older adolescents (≥16 years old) [21] and two studies included young and older adults (age range: 18–89 years) [27,29]. Of the 10 CVD studies, six considered both cats and dogs [20,22,25,26,27,29], while four focused solely on dogs [21,23,24,28]. Three studies focused on both all-cause mortality and any other type of CVD mortality [20,21,23], two studies focused solely on CVD mortality including stroke and myocardial infarction (MI) mortalities [25,27], two studies were on different CVD outcomes (e.g., coronary artery disease (CAD) or ischemic stroke) [22,24], one study was on CVD mortality and acute coronary syndrome (ACS) [26], one study was on CAD [29], and one study was on physical capacity in older adults who suffered a MI [28]. Operationalization of cat and/or dog ownership varied: one study did not describe how pet ownership was assessed [28], one study asked only one question about pet ownership [27], two studies used two questions on pet ownership [21,25], four studies used several items about pet ownership [20,22,26,29], and only two studies by the same lead author used a national dog registry [23,24].

Six of the CVD studies showed a reduced risk (positive effect) between all-cause mortality, CVD mortality, MI mortality, and/or coronary artery disease (CAD) risk and pet ownership [20,23,24,25,27,29]. Three of these studies included both cats and dogs [20,25,29] and three studies considered only dogs [23,24,28]. Two studies reported a protective effect specific to cat ownership [25,27]. Ogechi et al. (2016) found a decreased risk of dying from stroke among cat owners compared to dog owners [25]. Qureshi et al. (2009) reported an increased risk for CVD mortality in non-cat owners versus cat owners [27]. Two CVD studies reported mixed results (both positive and negative effects) for pet ownership and CVD mortality [22,27] and one study reported no association (effect) for dog ownership and all-cause mortality [21]. One CVD study reported an adverse association between cat ownership and cardiac death [26].

### 3.2. Obesity Studies

Sample sizes for the obesity studies ranged from *n* = 60 [32] to *n* = 473 [30] and all four studies included young, middle-aged, and older adults [30,31,32,33]. Three obesity studies focused on BMI and physical activity as outcomes [30,31,33], and one on weight loss, including fat percentage and waist circumference [32]. Two studies also reported on dietary intake [30,31], two reported on social support [31,33], and one reported on emotional stress [33]. Operationalization of pet ownership varied. Three studies used self-reported data to assess pet ownership but did not describe the question(s) asked [31,32,33] and one used a one-page questionnaire [30].

Two of the obesity studies were intervention studies [31,32] and two were observational studies [30,33]. Both observational studies reported a negative effect between pet ownership and obesity [30,33]. One study focused on both cats and dogs [30], and the other focused only on dogs [33]. Heuberger and Wakshlag (2011) reported that overweight in cats was associated with overweight (BMI > 25 kg/m^2^) in older (≥60 years old) cat owners and overweight in dogs was associated with overweight in older dog owners [30]. Stephens et al. (2012) found that although dog owners with overweight reported a greater sense of attachment with their pets (*r* = 0.29, *p* = 0.03), they also reported lower physical health (*r* = −0.20, *p* = 0.10) and social support (*r* = −0.27, *p* = 0.02) than normal-weight dog owners [33]. Both intervention studies reported no effect between dog ownership and obesity [31,32]. Kushner et al. (2006) conducted a weight loss intervention comparing people with obesity and their obese dogs to people with obesity and no pet (control group). At 12 months, there was no significant difference in weight loss between the intervention group (4.7%, standard deviation [SD] = 4.8%) and the control group (5.2%, SD = 6.2%) [31]. Niese et al. (2021) conducted two randomized clinical trials (one for humans, human clinical trial data reported here only, and one for dogs) for weight loss in two groups (owner-dog versus owner only). There was no statistical difference in mean weight loss between the owner-dog group (2.6%) and the owner only group (2.3%, *p* > 0.05) at the end of the intervention (8 weeks) [32].

### 3.3. Quality/Bias Assessment

Table 2 depicts the quality/bias assessment for the 14 studies. Generalizability was lacking across all studies. Studies were primarily with Non-Hispanic White populations. Other areas of weakness were quality of study outcomes and instrument validity. Cutoff points, detailed definitions (e.g., exercise is one type of physical activity and can consist of many different things), and how these CVD and obesity variables were collected were often not described in the 14 studies.

## 4. Discussion

This review systematically identified, evaluated, and summarized primary research with reported results on the human–animal relationship (cat and/or dog ownership) and CVD, obesity, and/or T2D outcomes in humans. Specifically, how cat and/or dog ownership was defined and measured, and what CVD, obesity, and/or T2D variables were used. Fourteen studies were included in this review, 10 of which were on CVD [20,21,22,23,24,25,26,27,28,29] and four on obesity [30,31,32,33]. No studies on T2D met the inclusion criteria. Most of the CVD studies focused on older adults who had previously experienced cardiovascular events [20,23,24,25,26,28,29]; hence, the findings of the influence of pet ownership on CVD events may not be generalizable. BMI and physical activity were the most common obesity outcomes [30,31,33]. However, how physical activity was measured differed greatly across the studies, which makes comparisons challenging. Some but not all measures were objectively obtained, cutoff levels for biomarkers (e.g., blood pressure) differed, and what constituted a behavior (e.g., physical activity) was often not well described, all of which make comparisons across studies difficult. Precise definitions and measurements would provide specificity to pet ownership research findings [34]. Others have found and reported similar findings [11,12,15]; this study adds more recent literature and a granular approach in terms of chronic disease outcomes across two of the most and common and preventable chronic diseases (CVD and obesity).

Findings on the association between cat and/or dog ownership and both CVD and obesity were mixed. This is contrary to other systematic reviews [12,35,36] that reported a decreased risk, often modest, between pet ownership and all-cause mortality and/or cardiovascular mortality. Further examination of these systematic reviews and meta-analyses show that the reported associations were restricted to subjects with post-ACS [12], cardiovascular mortality only [12,35], and reduced risk for cardiovascular mortality in the general population compared to people with CVD [35]. Given that these analyses were conducted according to specific parameters, including health status, the findings may not be comparable to those presented here. Furthermore, the studies included in this review varied in terms of sample size, CVD and obesity measures and outcomes, and characteristics of participants. These parameters may obfuscate the true results. Therefore, more research with experimental designs comparing both the general population and people with CVD and/or obesity as well as the use of standardized measures and outcomes are warranted.

Findings from these studies are further complicated by varied operationalization of pet ownership (the most common human–animal interaction). How pet ownership was defined and verified was wide-ranging and often not well described. Most of the studies collected only self-reported data about pet ownership [20,21,25,26,27,28,29,30,31,32,33]. Only two studies conducted by the same research team cross-referenced self-reported pet ownership with a national dog registry [23,24]. It is important to note that Mubanga et al. (2017, 2019) conducted their studies in Sweden [23,24], where a national health system with decentralized service delivery provides health care for residents, and this commitment to a universal health system by the federal government may have contributed to the establishment of a national dog registry. Although a national pet registry may not be feasible in all countries, efforts to introduce initiatives with this purpose in mind may improve both human and pet health. This is key since the One Health arena, the intersection and interconnection between people, animals, plants, and their shared environment, affects aspects of health and well-being for all [37]. A standardized and validated tool for pet ownership needs to be developed, which to the authors’ knowledge is lacking, so that time/duration, place, and other critical epidemiological characteristics can be collected for research. This tool could be implemented in existing USA-representative surveys such as the Behavioral Risk Factor Surveillance System and National Health Interview Survey as well as in other nationally representative surveys throughout the world.

Although a meta-analysis could not be completed because different types of studies were included, the rigor of each study was rated using a quality/bias assessment tool (Supplementary Table S1). The higher the summary score (summation of all scores), the higher the quality of the study. One shortcoming that nine studies (CVD: *n* = 5; obesity: *n* = 4) possessed was the lack of generalizability [20,26,27,28,29,30,31,32,33]. These studies lacked diversity, and their priority populations were typically older adults, White, and middle-/upper-middle class individuals. Only one study was conducted in Asia, specifically China, which reported a reduction in CAD disease risk among dog owners who had CAD [29]. More studies with Asian populations as well as global studies are needed to better understand the association between cat and/or dog ownership and CVD and obesity outcomes worldwide. Given that the study by Xie et al. (2017) focused only on CAD, additional CVD outcomes should also be studied. Finally, no study included Blacks or other racial/ethnic minorities. This is a huge gap in the literature. Studies including non-Whites are needed to fully understand the role of cat and/or dog ownership in CVD and obesity prevention and control in all populations.

Two other areas that are lacking in the literature, based on this review, are standardized reporting of health behaviors and the inclusion of intervention studies. Some studies included lifestyle behaviors (physical activity [20,25,29,30,31,33], diet [30,31], tobacco use [20,22,25,27,29], and alcohol use [20,21,25,29]) in their analyses, but these variables were not main outcomes, or their operationalization fully explained. Comparisons between the studies were tricky because of the variability in measurements used as well as omitted information (not reported in any study) such as the sizes of the pets (e.g., small dogs versus large dogs), dwellings (e.g., small apartments versus large homes), and locations (rural versus urban) of the owners, which may influence the time, duration, and intensity of walks or other forms of physical activity with pets. It is important to note that the relationship of pet owners with their pets may vary with the individual person as well as with the species of pet. Future studies should address lifestyle behaviors as they are modifiable risk factors that contribute to the onset of both CVD and obesity, and other chronic diseases. More intervention studies that investigate the interaction of pet ownership and lifestyle behaviors are needed.

This systematic review has some limitations. Study identification through literature searches was limited to two databases: PubMed and Web of Science. Nonetheless, more than 4500 potential studies were identified. This was a larger pool of potential studies than in previous reviews. Eligibility criteria restricted inclusion to primary research with English-language manuscripts published between the years 2000 and 2021. While the initial searches did yield a more-than-adequate number of manuscripts to consider for inclusion, there is an apparent need for more randomized clinical trials purposed for testing the established hypothesis concerning the association between cat and/or dog-ownership, CVD, and obesity as well as T2D.

## 5. Conclusions

There is an emerging, yet considerably incomplete, understanding of how human health is influenced by cat and/or dog ownership. The integration of beneficial associations of cat and/or dog ownership may make CVD and obesity prevention, mitigation, and treatment strategies more robust. A variety of measures need to be used in CVD and obesity research since onset of both are multifactorial and different research outcomes address different causes and health conditions. One way to improve the consistency of measurement is to develop specific human–animal interaction questions that can assess ownership through the life span and allow for prospective research on the role of pet ownership (an everyday human–animal interaction) in CVD and obesity. Inclusion of evidence-based cat and/or dog ownership information in CVD and/or obesity prevention can improve holistic care and optimize health outcomes. This work punctuates the findings of previous research [11,12,15]. Recommendations for the consistency of terms and measures used, inclusion of pet ownership questions and other forms of human–animal interactions in national health surveys, and more intervention research have not been heard. Given that prevention research within the One Health realm is a growing and promising area of research, these findings are a call to action. A call to action to One Health researchers to rethink how research is conducted, to stop working in silos, and to use interprofessional collaborations to expand research on human–animal relationships (i.e., pet ownership) and chronic disease, and to translate findings into practice.

## Figures and Tables

**Table 1:** Summary of included studies (*n* = 14) and pet ownership effect on CVD and obesity outcomes.

Study	Study Sample	Pet Ownership	Outcome Measure(s)	Reported Effect
CVD				
Chowdhury et al., 2017 [20]	older adults (65–84 years old) *n* = 4039 (never owners = 549, current owners = 1456, previous owners = 2034)	cat, dog	all-cause mortality, CVD ^1^ mortality	positive
Ding et al., 2018 [21]	adults (≥16 years old) *n* = 59,352 (dog owners = 17,071, non-owners = 42,281)	dog	all-cause mortality, CVD mortality	no effect
Krittanawong et al., 2020 [22]	adults *n* = 10,905 (dog owners = 4577, cat owners = 6328)	cat, dog	CAD ^2^, heart failure, DM ^3^, stroke, systemic hypertension	mixed
Mubanga et al., 2017 [23]	adults (40–80 years old) *n* = 3432,153	dog	all-cause mortality, CVD mortality, acute MI ^4^	positive
Mubanga et al., 2019 [24]	adults (40–85 years old) cute MI: *n* = 181,696 ischemic stroke: *n* = 154,617	dog	acute MI, ischemic stroke	positive
Ogechi et al., 2016 [25]	adults (≥16 years old) *n* = 3964	cat, dog	CVD mortality, stroke mortality	positive
Parker et al., 2010 [26]	patients hospitalized with ACS ^5^ *n* = 424 (12 withdrew/unable to contact)	cat, dog	ACS readmission, CVD mortality	negative
Qureshi et al., 2009 [27]	adults (18–74 years old) *n* = 4435	cat, dog	MI mortality, CVD mortality	mixed
Ruzic et al., 2011 [28]	older adults *n* = 59 males (owners = 29, non-owners = 30)	dog	physical capacity maximal workload, heart rate, BP ^6^	positive
Xie et al., 2017 [29]	adults (30–89 years old) *n* = 561	cat, dog	CAD	positive
Obesity				
Heuberger and Wakshlag, 2011 [30]	adults (≥17 years old) *n* = 473	cat, dog	BMI, dietary intake	negative
Kushner et al., 2006 [31]	adults (21–65 years old) *n* = 92	dog	BMI ^7^, PA ^8^	no effect
Niese et al., 2021 [32]	adults with BMI ≥ 25 *n* = 60 (owner-dog: *n* = 29, owner only: *n* = 31)	dog	weight loss	no effect
Stephens et al., 2012 [33]	adults (≥18 years old) *n* = 75	dog	BMI, PA, stress, social support	negative

^1^ CVD: cardiovascular disease; ^2^ CAD: coronary artery disease; ^3^ DM: diabetes mellitus; ^4^ MI: myocardial infarction; ^5^ ACS: acute coronary syndrome; ^6^ BP: blood pressure; ^7^ BMI: body mass index; ^8^ PA: physical activity.

**Table 2:** Quality/bias assessment of studies (*n* = 14) that met inclusion criteria.

Study	Degree of Pertinence (Fitness)	Quality of Study Outcomes	Instrument Validity	Generalizability	Summary Score (Range: 4–12)
Cardiovascular Disease					
Chowdhury et al. 2017 [20]	2	3	1	2	8
Ding et al. 2018 [21]	3	3	2	3	11
Krittanawong et al. 2020 [22]	3	3	3	3	12
Mubanga et al. 2017 [23]	3	3	3	3	12
Mubanga et al. 2019 [24]	3	3	3	3	12
Ogechi et al. 2016 [25]	3	3	2	3	11
Parker et al. 2010 [26]	3	3	3	2	11
Qureshi et al. 2009 [27]	3	3	3	2	11
Ruzic et al. 2011 [28]	1	1	1	1	4
Xie et al. 2017 [29]	3	3	1	1	8
Obesity					
Heuberger et al. 2011 [30]	1	1	1	1	4
Kushner et al. 2006 [31]	3	3	2	1	9
Niese et al. 2021 [32]	2	3	2	1	8
Stephens et al. 2012 [33]	2	3	2	1	8

## Supplementary Materials

The following are available online at https://www.mdpi.com/article/10.3390/vetsci8120333/s1, Supplementary Table S1: Quality and bias assessment parameters for included studies; Supplementary Table S2: Studies (*n* = 14) that met all inclusion criteria.
