# Supplementary material for: Cat and/or Dog Ownership, Cardiovascular Disease, and Obesity: A Systematic Review
Source: Vet Sci. 2021 Dec 17;8(12):333. doi: 10.3390/vetsci8120333 (PMC8706375; doi:10.3390/vetsci8120333)
Supplement: Supplementary file 1 [file vetsci-08-00333-s001.zip › vetsci-1458175-supplementary.pdf]

Supplementary Table S1. Quality and Bias Assessment Parameters for Included Studies.

Assessment Parameter	Score & Rating
Degree of Pertinence (Fitness)	3: The study focuses on, and directly addresses, one or more of this review's focal chronic disease (i.e., CVD, T2D, or obesity), and clearly defines pet ownership. It further identifies participants and/or groups using established biomarkers or components of CVD, T2D, or obesity.<br>2: The study focuses on, and directly addresses, one or more of this review's focal chronic disease (i.e., CVD, T2D, or obesity), and clearly defines pet ownership.<br>1: The study directly addresses one or more of this review's focal chronic diseases (i.e., CVD, T2D, or obesity).
Quality of outcomes	3: The study's outcome measures appropriately identify and describe variables of interests; outcomes are clearly defined and were objectively obtained.<br>2: The study's outcome measures appropriately identify and describe variables of interests; either outcomes are not clearly defined or were subjectively obtained (e.g., self-report).<br>1: The study's outcome measures appropriately identify and describe variables of interests; the outcomes are not clearly defined and/or were subjectively obtained (e.g., self-report).
Instrumentation Validity	3: Study uses a previously validated instrument for data collection or study sources data from a previously conducted survey that used a validated survey instrument.<br>2: Study uses elements from, or adapts, a validated instrument for data collection.<br>1: Study uses an unvalidated instrument for data collection, or the author does not report whether or not the instrument has been validated.
Degree of Generalizability	3: Generalizable to the national population.<br>2: Limited generalizability (i.e., generalizable to localized populations and/or population segments).<br>1: Not generalizable.
Summary score (sum of all scores)	12: High quality<br>8: Medium quality<br>4: Lesser quality

Supplementary Table S2. Studies (n=14) that Met All Inclusion Criteria.

Study	Purpose	Description	Measures	Outcomes
**Cardiovascular Disease (CVD)**				
Chowdhury et al., 2017 [20]<br><br>Pet ownership and survival in the elderly hypertensive population	Assess the association of pet ownership, all-cause mortality, and cardiovascular mortality over a long-term follow-up (~11 years) among elderly hypertensive participants	Country<br>Australia: 5 states (family practice)<br><br>Study design<br>comparative outcome trial (subsample of Second Australian National Blood Pressure)<br><br>Study sample<br>65-84 years old with treated hypertension<br>N=4,039 (never owners=549, current owners=1,456, previous owners=2,034)<br><br>Time period<br>1995-1997, mid-2000s	Independent variable<br>pet-ownership: cat, dog, other<br><br>Dependent variable<br>all-cause mortality, CVD mortality<br><br>CVD measures<br>BMI [1], SBP [2], DBP [3], total cholesterol, HDL [4], LDL [5], eGFR [6], PA [7], ACE [8]<br><br>Pet ownership<br>5-item instrument: currently owned or had ever owned, type & number of pets, lived inside or outside home, walked dog (daily, most days, rarely or never)	All-cause mortality<br>16% reduction in previous owners, 11% reduction in current owners vs never-owners, p=.06<br><br>CVD mortality<br>22% reduction in previous owners, 26% reduction in current owners vs never-owners, p=.05

Ding et al., 2018 [21]<br><br>Dog ownership and mortality in England: a pooled analysis of six population-based cohorts	Examine the association of dog ownership with all-cause mortality and CVD mortality	Country<br>England<br><br>Study design<br>multistage stratified probability design (Health Surveys for England)<br><br>Study sample<br>≥16 years old<br>N=59,352 (dog owners=17,071, non-owners=42,281)<br><br>Time period<br>1995, 1996, 1997, 2001, 2002, 2004	Independent variable<br>pet-ownership: dog<br><br>Dependent variable<br>all-cause mortality, CVD mortality<br><br>CVD measures<br>diabetes mellitus, smoking status, alcohol consumption<br><br>Pet ownership<br>head of household asked: *"Do you keep any household pets inside your house/flat?"*, type of pet	All-cause mortality<br>hazard ratio=1.03, 95% confidence interval (CI)=0.98-1.09 (no significant association)<br><br>CVD mortality<br>hazard ratio=1.07, 95% CI=0.96-1.18 (no significant association)
Krittanawong et al., 2020 [22]<br><br>Pet ownership and cardiovascular health in the U.S. general population	Assess the association between pet ownership (dog or cat) and CVD health in a heterogeneous population	Country<br>USA<br><br>Study design<br>longitudinal prospective (National Health and Nutrition Examination Survey [NHANES])<br><br>Study sample<br>N=10,905 (dog owners=4,577, cat owners=6,328)<br><br>Time period<br>1999-2016	Independent variable<br>pet-ownership: cat, dog<br><br>Dependent variable<br>CAD [9], heart failure, diabetes mellitus, stroke systemic hypertension<br><br>CVD measures<br>SBP [2], DBP [3], total cholesterol, HDL [4], LDL [5], TG [10], hyperlipidemia, Framingham risk score, cigarette smoking<br><br>Pet ownership<br>4 items: *"Dog in house now. Dog in house, last 12 months. Cat in house now. Cat in house, last 12 months."*	CAD [9]<br>either dog or cat: odds ratio (OR)=0.97 (95% CI=0.72-1.29, p=0.82)<br><br>Heart failure<br>either dog or cat: OR=1.05 (95% CI=0.77-1.43, p=0.73)<br><br>Diabetes mellitus<br>either dog or cat: OR=0.87 (95% CI=0.74-1.03, p=0.08)<br><br>Stroke<br>either dog or cat: OR=0.83 (95% CI=0.61-1.12, p=0.22)<br><br>Systemic hypertension<br>dog & cat: OR=0.69 (95% CI=0.54-0.89, p=0.01)

Mubanga et al., 2017 [23]<br><br>Dog ownership and the risk of cardiovascular disease and death - a nationwide cohort study	Assess the association of dog ownership with CVD and mortality in a registered-based prospective nation-wide cohort with up to 12 years of follow-up	Country<br>Sweden<br><br>Study design<br>Longitudinal prospective (Register of the Total Population [RTP], Swedish Twin Registry [STR]: Screening Across the Lifespan Twin study)<br><br>Study sample<br>40-80 years<br>RTP: N=3,432,153<br>STR: N= 34,202<br><br>Time period<br>RTP: 1/1/2001-12/31/2012<br>STR: 1/1/2001-12/31/2014	Independent variable<br>pet-ownership: dog<br><br>Dependent variable<br>all-cause mortality, acute MI [11], heart failure, ischemic stroke, hemorrhagic stroke, composite CVD incidents, CVD mortality<br><br>CVD measures<br>N/A [12]<br><br>Pet ownership<br>registered or having a partner registered as a dog owner in either of two national dog registers	All-cause mortality (RTP)<br>hazard ratio=0.80, 95% CI=0.79-0.82<br><br>Acute MI [11] mortality (RTP)<br>hazard ratio=0.97, 95% CI=0.95-0.99<br><br>CVD mortality (RTP)<br>hazard ratio=0.77, 95% CI=0.73-0.80
Mubanga et al., 2019 [24]<br><br>Dog ownership and survival after a major cardiovascular event: a register-based prospective study	Assess the association of dog ownership with survival and recurrent events after a major cardiovascular event in a large prospective, nationwide register-based cohort study	Country<br>Sweden<br><br>Study design<br>Longitudinal prospective (Swedish National Patient Register)<br><br>Study sample<br>40-85 years<br>acute MI [11]: n=181,696 (10,287 dog owners)<br>ischemic stroke: n=154,617 (7,344 dog owners)<br><br>Time period<br>1/1/2001-12/31/2012<br>no event 1999-2001	Independent variable<br>pet-ownership: dog<br><br>Dependent variable<br>acute MI [11], ischemic stroke<br><br>CVD measures<br>use of medication for hypertension, dyslipidemia, diabetes mellitus<br><br>Pet ownership<br>registered as a dog owner in either of two national dog registers	Acute MI [11] mortality<br>living alone: hazard ratio=0.67, 95% CI=0.61-0.75<br>living with partner/child: hazard ratio=0.85, 95% CI=0.80-0.90<br><br>Ischemic stroke mortality<br>living alone: hazard ratio=0.73, 95% CI=0.66-0.80<br>living with partner/child: hazard ratio=0.88, 95% CI=0.83-0.93

Ogechi et al., 2016 [25]<br><br>Pet ownership and the risk of dying from cardiovascular disease among adults without major chronic medical conditions	Examine the association between pet ownership and CVD risk in people without established CVD	Country<br>USA<br><br>Study design<br>longitudinal prospective (NHANES III)<br><br>Study sample<br>≥50 years without chronic disease diagnosis at baseline<br>N=3,964<br><br>Time period<br>1988-1994, 2006	Independent variable<br>pet-ownership: cat, dog, other<br><br>Dependent variable<br>CVD mortality, acute ischemia, stroke, hypertension<br><br>CVD measures<br>PA [7] level, BMI [1], cigarette smoking, alcohol consumption<br><br>Pet ownership<br>2-part questionnaire: *"Does a pet live here? If yes, what type of pet is it?"*	CVD mortality<br>hazard ratio=0.69, 95%, CI=0.45-1.07 among women (weak association – cat ownership)<br><br>Stroke mortality<br>hazard ratio=0.54, 95%, CI=0.28-1.01 among women (weak association – cat ownership)
Parker et al., 2010 [26]<br><br>Survival following an acute coronary syndrome: a pet theory put to the test	Re-examine findings from previous studies reporting an association between pet ownership and coronary artery disease survival at 12 months in patients hospitalized with acute coronary syndrome	Country<br>Australia: Sydney (large teaching hospital) & surrounding districts<br><br>Study design<br>longitudinal prospective<br><br>Study sample<br>patients hospitalized with ACS [13], including MI [11] & unstable angina<br>N=424 (12 withdrew/unable to contact)<br><br>Time period<br>2-year period	Independent variable<br>pet-ownership: cat, dog, bird<br><br>Dependent variable<br>ACS [13] readmission, CVD mortality<br><br>CVD measures<br>diabetes on admission, CVA [14] or TIA [15] on admission, LVEF [16], CABG [17], post-ACS [13] onset depression<br><br>Pet ownership<br>participants asked if they had a pet in their household, type of pet, whether that pet belonged to them	Univariate analyses<br>pet owners (those who had a household pet at baseline) were more likely to experience an ACS [13] readmission or cardiac-related death over 12 months (22.2%) than non-pet owners, 13.6%, X^2= 4.7, df=1, p=0.030<br><br>Multivariate analyses<br>no difference in rates of ACS [13] readmission & cardiac death for dog owners (21.9%) vs non-dog-owners (16.3%, X^2=1.3, df=1, p= 0.249)<br><br>difference in rates of ACS readmission & cardiac death for cat owners (27.3%) vs non-cat owners (16.2%) approached significance (X^2=3.25, df=1, p= 0.071)

Qureshi et al., 2009 [27]<br><br>Cat ownership and the risk of fatal cardiovascular diseases. Results from the second National Health and Nutrition Examination Study mortality follow-up study	Determine the effect of pet ownership on fatal cardiovascular events in a nationally representative cohort followed for mean period of 13.4±3.6 years	Country<br>USA<br><br>Study design<br>longitudinal prospective (NHANES II follow-up study)<br><br>Study sample<br>18-74 years old<br>N=4,435<br><br>Time period<br>1976-1980	Independent variable<br>pet-ownership: cat, dog<br><br>Dependent variable<br>all-cause mortality, MI mortality, CVD (MI or stroke) mortality, stroke<br><br>CVD measures<br>SBP [2], serum cholesterol level, BMI [1], diabetes mellitus, cigarette smoking<br><br>Pet ownership<br>owned or currently owned a cat or dog	MI mortality<br>lower relative risk in previous cat owners than non-cat owners (RR=0.63, 95% CI=0.44-0.88)<br><br>CVD mortality<br>increased risk for death due to CVD in non-cat owners than cat owners (RR=0.74, 95% CI=0.55-1.0)
Ruzic et al., 2011 [28]<br><br>Regular dog-walking improves physical capacity in elderly patients after myocardial infarction	Determine the influence of regular (every day) dog-walking on the physical capacity in patients during the first year after myocardial infarction	Country<br>Croatia<br><br>Study design<br>longitudinal prospective & controlled<br><br>Study sample<br>older adult patients who were hospitalized for MI [11]<br>N=59 males (owners=29, non-owners=30)<br><br>Time period<br>12 months	Independent variable<br>dog-walking (3x15 minutes/day), walking (30 minutes/day)<br><br>Dependent variable<br>physical capacity from Bruce bicycle-ergometry test 1-year after MI [11] (workload, heart rate, BP [18])<br><br>CVD measures<br>workload, heart rate, BP [18]<br><br>Pet ownership<br>not described	Physical capacity<br>maximal workload higher in the dog-walking group (72.5±10.75) than in non-dog walking group (67.6±11.6, p<0.05)<br><br>maximal heart rate and maximal BP not significantly different at the beginning or the end of the intervention for either group

Xie et al., 2017 [29]<br><br>Association between pet ownership and coronary artery disease in a Chinese population	Assess the association between pet ownership and CAD [9] patients in a Chinese population	Country<br>China<br><br>Study design<br>retrospective<br><br>Study sample<br>30-89 years old, suffered from typical or atypical chest pain or abnormal ST segment, T wave changes, & admitted for coronary arteriography<br>N=561 (males=376, females=185, CAD [9] patients=378, non-CAD [9] patients=183)<br><br>Time period<br>October 2015-May 2016	Independent variable<br>pet-ownership: cat, dog<br><br>Dependent variable<br>CAD [9] diagnosis (Judkins arteriography technique: stenosis of ≥50% in any of major coronary arteries)<br><br>CVD measures<br>BMI [1], hypertension, hyperlipidemia, PA [7], diabetes mellitus, smoking status, drinking status, family history of CAD [9]<br><br>Pet ownership<br>questionnaire: current cat owner, current dog owner, current cat & dog owner, pet owners asked: *"How long have you kept pets? How long do you stay with your pets per day? When did you start to keep pets?"*	CAD [9] diagnosis<br>decreased CAD [9] risk among pet owners (OR=0.504; 95% CI=0.310–0.819)<br><br>reduced CAD [9] risk among dog owners (OR=0.420, 95% CI=0.242–0.728) compared with cat owners (OR=0.738; 95% CI=0.240–2.266) & compared with cat & dog owners (OR=1.052; 95% CI=0.330–3.355)<br><br>duration of pet ownership is inversely related to CAD [9] risk (p for trend=0.008) & time playing with pets per day (p for trend=0.001)

**Obesity**				
Heuberger & Wakshlag, 2011 [30]<br><br>Characteristics of ageing pets and their owners: dogs v. cats	Examine differences in diet and lifestyle between cat and dog owners and their pets	Country<br>USA<br><br>Study design<br>cross-sectional<br><br>Study sample<br>>17 years old, pet owner<br>N=473<br><br>Time period<br>not described	Independent variable<br>pet ownership: cat, dog<br><br>Dependent variable<br>PA [7] (exercise), dietary intake, fast food consumption, BMI [1], health status<br><br>Obesity measures<br>BMI, health status (not described), dietary intake (not described), PA [7] (not described)<br><br>Pet ownership<br>1-page questionnaire	BMI [1]<br>overweight (BMI>25 kg/m^2) in dog owners was inversely related to age of dog ($p<0.01$)<br><br>younger dog owners with obesity more likely to have a dog with overweight ($p<0.04$)<br><br>Dietary intake<br>fast food consumption by dog owners was inversely related to age of dog ($p<0.05$)<br><br>PA [7]<br>exercise by dog owners was inversely related to age of dog ($p<0.05$)

Kushner et al., 2006 [31]<br><br>The PPET study: People and pets exercising together	Assess the effectiveness of a combined people and pets (PP) weight loss program where both human participants and companion dogs were obese	Country<br>USA<br><br>Study design<br>Prospective, controlled trial<br><br>Study sample<br>21-65 years old with BMI>25kg/m^2 in good health<br>N=92<br><br>Time period<br>1-year	Independent variable<br>pet-ownership: dog<br><br>Dependent variable<br>BMI [1] (weight loss), PA [7], social support & exercise readiness scores, social support (qualitative)<br><br>Obesity measures<br>BMI [1], PA [7] (20-30 minutes/day) recorded in log, ~1400 calories/day recorded in log, social support & exercise readiness scores<br><br>Pet ownership<br>self-reported, confirmed in telephone screen	BMI [1]<br>no significant difference in mean percentage weight loss at 12 months (LOCF) for dog owners vs non-owners<br><br>PA [7]<br>dog owners: mean total PA [7] increased 39% (2.8 hr/wk at baseline to 3.9 hrs/wk at 12 months, p<0.05)<br>non-owners: mean total PA [7] increased 87% (1.9 hr/wk at baseline to 3.5 hrs/wk, p<0.05)<br>dog owners : two-thirds of total PA [7] spent with as dog-related activity<br><br>Social support & exercise readiness scores<br>No difference between dog owners & non-owners

Niese et al., 2021 [32]<br><br>Evaluating the potential benefit of a combined weight loss program in dogs and their owners	Evaluate the potential mutual effects of a weight loss program for both dogs and owners on each other	Country<br>Netherlands<br><br>Study design<br>2 randomized clinical trials (human & dog) each with 2 arms (human clinical trial: owner-dog & owner only)<br><br>Study sample<br>adult with BMI≥25 and able to walk for at least 1 hour/day<br>N=60 (owner-dog: n=29, owner only: n=31)<br><br>Time period<br>8 weeks	Independent variable<br>1-day food diary, steps per day, lifestyle knowledge, attitudes, & beliefs, diet & physical activity behaviors<br><br>Dependent variable<br>weight loss<br><br>Obesity measures<br>body weight, fat percentage, waist circumference<br><br>Pet ownership<br>self-reported (not described)	Weight loss<br>2.6% mean weight loss in owner-dog group vs 2.3% mean weight loss in owner only, $p>0.05$

Stephens et al., 2012 [33]<br><br>Health perceptions and levels of attachment: owners and pets exercising together	Describe baseline characteristics of dog owners' physical health and their levels of pet attachment<br><br>Owners & Pets Exercising Together (OPET) trial	Country<br>USA<br><br>Study design<br>cross-sectional<br><br>Study sample<br>≥18 years old able to engage in regular PA and owned a dog (at least 2 years old)<br>N=75<br><br>Time period<br>not described	Independent variable<br>BMI [1], perceived physical health<br><br>Dependent variable<br>PA [7], stress, social support, level of attachment with dog<br><br>Obesity measures<br>BMI [1], PA [7], stress, social support<br><br>Pet ownership<br>self-reported (not described)	Perceived health<br>BMI [1] inversely correlated with perceived physical health (r=-0.20, p=0.10)<br><br>PA [7]<br>no correlation between BMI [1] and PA[7]<br>perceived physical health correlated with PA [7] (r=0.26, p=0.03)<br><br>Stress<br>no correlation between BMI [1] and stress<br><br>Social support<br>BMI [1] inversely correlated with social support (r=-0.27, p=0.02)<br><br>Attachment with dog<br>BMI [1] correlated with attachment to dog (r=0.29, p=0.03)

[1] BMI: body mass index, [2] SBP: systolic blood pressure, [3] DBP: diastolic blood pressure, [4] HDL: high-density lipoprotein cholesterol, [5] LDL: low-density lipoprotein cholesterol, [6] eGFR: kidney glomerular filtration rate, [7] PA: physical activity, [8] ACE: angiotensin converting enzyme inhibitor, [9] CAD: coronary artery disease, [10] TG: triglycerides, [11] MI: myocardial infarction, [12] N/A: not applicable, [13] ACS: acute coronary syndrome, [14] CVA: cerebrovascular accident, [15] TIA: transient ischemic attack, [16] LVEF: left ventricular ejection fraction, [17] CABG: coronary artery bypass graft, [18] BP: blood pressure
